# A Large Root Phenome Dataset Wide-Opened the Potential for Underground Breeding in Soybean

**DOI:** 10.3389/fpls.2021.704239

**Published:** 2021-08-05

**Authors:** Ki-Seung Kim, Se-Hun Kim, Jaeyoung Kim, Pooja Tripathi, Jeong-Dong Lee, Yong Suk Chung, Yoonha Kim

**Affiliations:** ^1^Department of Innovative Technology, FarmHannong, Ltd., Nonsan, South Korea; ^2^Department of Applied Biosciences, Kyungpook National University, Daegu, South Korea; ^3^Department of Plant Resources and Environment, Jeju National University, Jeju, South Korea

**Keywords:** link average diameter, link average branching angle, phenomics, projected area, WinRHIZO

## Abstract

The root is the most critical plant organ for water and nutrient acquisition. Although the root is vital for water and nutrient uptake, the diverse root characters of soybean still need to be identified owing to the difficulty of root sampling. In this study, we used 150 wild and 50 cultivated soybean varieties to collect root image samples. We analyzed root morphological traits using acquired-image. Except for the main total length (MTL), the root morphological traits for most cultivated and wild plants were significantly different. According to correlation analysis, the wild and cultivated plants showed a significant correlation among total root length (TRL), projected area (PA), forks, total lateral length (TLL), link average diameter, and MTL. In particular, TRL was highly correlated with PA in both cultivated (0.92) and wild (0.82) plants compared with between MTL (0.43 for cultivated and 0.27 for wild) and TLL (0.82 for cultivated and 0.52 for wild). According to principal component analysis results, both plants could be separated; however, there was some overlap of the traits among the wild and cultivated individuals from some regions. Nevertheless, variation among the cultivated plants was higher than that found in the wild plants. Furthermore, three groups, including MTL, TLL, and the remaining traits, could explain all the variances.

## Introduction

Soybean (*Glycine max* L.) is regarded as a significant worldwide crop owing to its nutritional value (Kim et al., 2015). According to Nawaz et al. (2018), soybean is classified into 28 species under 2 subgenera. Among them, *G. max* and *G. soja* Sieb. and Zucc are consumed as food by humans and livestock (Nawaz et al., 2018).

*Glycine max* (cultivated soybean) is an annual legume with white to purple–pink flowers and trifoliate leaves and an extensive taproot system; most of the taproot system is in the top 15 cm soil layer (Chaturvedi et al., 2011). Cultivated soybean was domesticated from its annual relative *G. soja* (wild soybean) (Carter et al., 2004). Soybean is native to East Asia, and it is widely cultivated for its edible beans in Korea, China, Japan, and Russia (Jeong et al., 2019). Soybean has numerous uses (Multilingual Multiscript Plant Name Database: Retrieved Feb 16, 2012). It is an economically important legume crop that provides food and animal feed (Graham and Vance, 2003). In China, which is the world’s largest soybean genetic diversity reservoir (Li et al., 2008), 23,587 soybean landraces have been collected.

Several pieces of evidence, including proteomics, genomics, and cytological traits (Wang et al., 2016), suggest that wild soybean is the progenitor species of cultivated soybean. Moreover, wild soybean is widely distributed in East Asia, including Korea, China, Japan, and the Russian Far East. Moreover, it grows well in diverse areas, including agricultural fields, lakesides, marshlands, and riverbank, within any nation (Lu, 2004; Wang et al., 2016). Thus, it is well acclimated to various environmental conditions because of its broad regional adaptability (Nawaz et al., 2018). The wild soybean contains enriched characters known as gene banks for the species (Li et al., 2017; Nawaz et al., 2018). Hence, various traits have been comprehensively characterized, focusing on the above-ground organs of wild soybean.

The root is essential for water acquisition and nutrient absorption across the entire life of a plant (Zhao et al., 2017). It participates in nutrient cycling and soil formation stabilization *via* its interaction with soil organisms (Bardgett et al., 2014; Faucon et al., 2017). Thus, there is a considerable potential for breeding better cultivars by understanding root morphological traits associated with plant growth and development. This makes the root important for identifying crucial traits. However, the root system has not been studied in detail compared with the above-ground organs of the plants because of difficulty in phenotyping them. In addition, manual measuring of the root traits is time-consuming, laborious, and inaccurate in a fully grown plant (Costa et al., 2001; French et al., 2009; Lobet et al., 2011). Hence, phenotyping of the architecture of the root system is performed primarily under controlled laboratory conditions at early growth stages, although “shovelomics” has also been used for both field breeding and quantitative genetics (Colombi et al., 2015). However, the shovelomic method measures the ground nodal root (crown root) phenotypes, disregarding the internal root system despite its enormous impact on plant growth (York and Lynch, 2015).

Due to developments in imaging technology, high-resolution images can be easily captured using a compact camera (Chung et al., 2017). Many efforts have been employed to apply image-based phenotyping for high-throughput phenotyping in agricultural research fields (Vasseur et al., 2018; Kim et al., 2019). In particular, many imaging analysis software were launched to determine root growth and development (Judd et al., 2015; Richard et al., 2015). In this context, WinRHIZO root-scanning software (Regent Instruments Inc., Ottawa, ON, Canada) is unique for measuring several root morphologies, including total root length (TRL), average root diameter, projected area (PA), number of tips, and forks (FK) (Pornaro et al., 2017). This software comprises two parts: one is the scanner, which acquires images, and the other is the analysis software in the computer. This equipment can quickly analyze several root traits *via* the acquired two-dimensional (2D) images (Arsenault et al., 1995). Hence, it has been used to identify root morphology and architecture (Wang and Zhang, 2009; Pang et al., 2011; Tajima and Kato, 2013; Pornaro et al., 2017). The present study aimed to screen root morphology to evaluate the variation in root traits among the cultivars and wild germplasm of soybean.

## Materials and Methods

### Plant Materials and Growth Conditions

We used 200 soybeans (150 wild soybean accessions and 50 cultivars; Supplementary Table 1) to evaluate root characteristics in soybean. Each accession comprised three plants. We used polypropylene (PVC) pipes as pots to collect root samples without damage. The size of the PVC pot was 6 (diameter) × 40 cm (height). The seeds were sterilized with 70% ethanol and then washed with double distilled water thoroughly. Three experimental replicates were performed, and a single plant was used as one replication for root analysis. We planted two sterilized seeds on the PVC pots containing horticulture soil (Tobirang, Baekkwang Fertility, South Korea) to reduce the chances of the seeds not germinating. Then, we placed the pipes in a greenhouse that is located at the research farm in Kyungpook National University. After germination, only one soybean plant was used to collect the root sample.

### Phenotypic Data Collection

#### Root Data Collection

**FIGURE 1:**
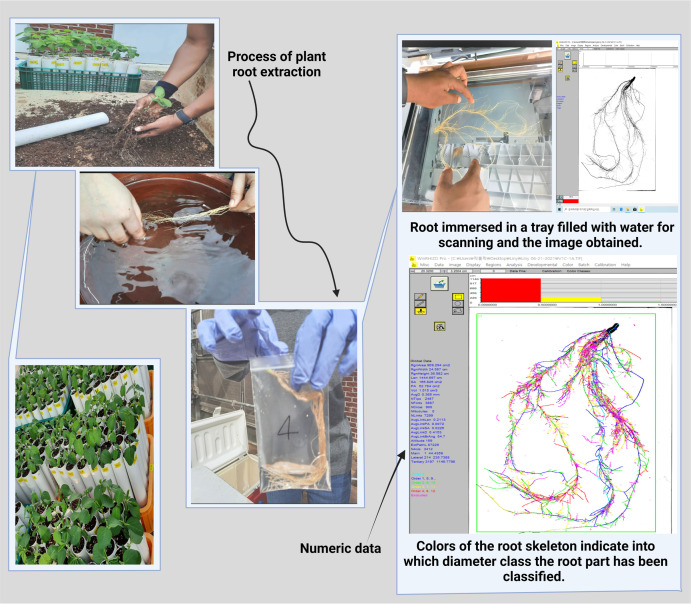
Process of root analysis for morphological data collection.

We collected root samples when the soybean plant reached the V1 growth stage with fully developed leaves at the unifoliate node (Jones et al., 1991). We carefully removed the stopper from the bottom of the PVC pipes to minimize root damage or loss. Then, we removed all soil and soybean roots from the pipes. We carefully transferred the soil and soybean root samples to a sieve and separated the soil from the roots using fresh water. The clean root samples were transferred to a plastic bag containing distilled water until image analysis to prevent the root from drying. We analyzed the root morphological traits using a 2D image captured by a scanner (Expression 12000XL, Epson, Japan). The clean root samples were placed on a transparent tray (30 cm × 20 cm), and tap water was poured carefully until the root samples floated. Images of the floating root samples were captured using the scanner. The root images were added to WinRHIZO pro software (Regent Instruments Inc., Canada) for root morphological data analysis (Figure 1). We annotated and analyzed only the root area in the original image to avoid sampling error data. Supplementary Table 2 describes the root morphological traits obtained with the WinRHIZO software.

### Statistical Analysis

#### One-Way Analysis of Variance

We performed two experiments: the first experiment identified the root traits among 150 wild plants and the second experiment evaluated the root traits in 50 cultivated plants. Hence, a total of 200 soybean genotypes (150 wild and 50 cultivated) were used for identifying root characteristics. To determine differences among soybean accessions and to verify that there is no effect from replications, all data were transformed into square roots for normalizing the distribution of data. One-way analyses of variance (SAS v.9.4; SAS, Gary, NC, United Sates) were performed twice for replications and genotypes. The equation is as follows:

(1) $$Y_{ij}=\mu +\tau_{j}+e_{ij},$$

Where, *Y*_*ij*_ is the *i*^*th*^ and *j*^*th*^ quantified phenotype observations, *i* is the index regarding the observations of the phenotype in the entire dataset, *j* is the index of over replication of genotype groups, τ_*ij*_ is the *i*^*th*^ and *j*^*th*^ effect of replications or genotypes, and *e* is the error.

#### Kruskal–Wallis Rank-Sum Test and Spearman’s Rank Correlation Test

The Kruskal–Wallis rank-sum test was performed using a raw dataset of 200 genotypes to demonstrate differences in the phenotypes of wild and cultivated soybean cultivars. Moreover, the Spearman’s rank correlation test was performed to identify correlations among the phenotypes of the cultivars. The R programming language was used for performing these tests (R v. 4.0.4; R Foundation for Statistical Computing, Vienna, Austria).

#### Principal Component Analysis Plot Analysis

Principal component analysis (PCA) was performed for the same 200 soybean accessions, (150 wild and 50 cultivated cultivars) in R. The analysis was performed using five traits that showed significant differences between the wild and cultivated cultivars [TRL, PA, FK, main total length (MTL), and total lateral length (TLL)]. The PCA plot was generated through the R packages “devtools” and “ggbiplot” for choosing the first and second principal components.

## Results

### Variations Among the Cultivated and Wild Plants

Both the cultivated and wild plants were significantly different from one another with respect to the root morphological traits obtained from image analysis, except for MTL (0.060) among the cultivated plants (Table 1). We could infer that MTL was selected as a direction or breeding goal in the cultivated plants during the domestication process of soybean. The *p*-value of MTL for the wild plants was relatively higher (0.008) than that of the rest of the traits (<0.001 for all others), suggesting that MTL among the wild plants was somewhat selected compared with other characteristics. Likewise, the relatively high *p*-value of TLL (0.0126) in the cultivated plants might suggest the same domestication pattern.

**TABLE 1:** Analysis of variance for the wild (*n* = 150) and cultivated soybean (*n* = 50).

Wild													
Effects	df	TRL_sqrt		PA sqrt		FK_sqrt		MTL_sqrt		TLL_sqrt		LAD_sqrt	
		*F* value	Pr(>*F*)	*F* value	Pr(>*F*)	*F* value	Pr(>*F*)	*F* value	Pr(>*F*)	*F* value	Pr(>*F*)	*F* value	Pr(>*F*)
Rep	2	3.376	0.068	302.59	< 0.001***	10.342	0.002**	1.230	0.269	0.193	0.661	329.586	< 0.001***
Genotype	149	14.465	< 0.001***	13.04	< 0.001***	8.177	< 0.001***	1.492	0.008**	3.011	< 0.001***	1.854	< 0.001***

		**Cultivated**											
		**TRL_sqrt**		**PA sqrt**		**FK_sqrt**		**MTL_sqrt**		**TLL_sqrt**		**LAD_sqrt**	

Rep	2	0.128	0.722	37.133	< 0.001***	22.148	< 0.001***	11.949	0.001***	5.426	0.0219*	76.855	< 0.001***
Genotype	49	19.084	< 0.001***	9.577	< 0.001***	6.677	< 0.001***	1.453	0.060	1.708	0.0126*	2.382	< 0.001***

TRL, total root length; PA, projected area; FK, forks; MTL, main total length; TLL, total lateral length; LAD, link average diameter. * <0.05, ** <0.01, *** <0.001.

### Comparison Between the Cultivated and Wild Plants

**FIGURE 2:**
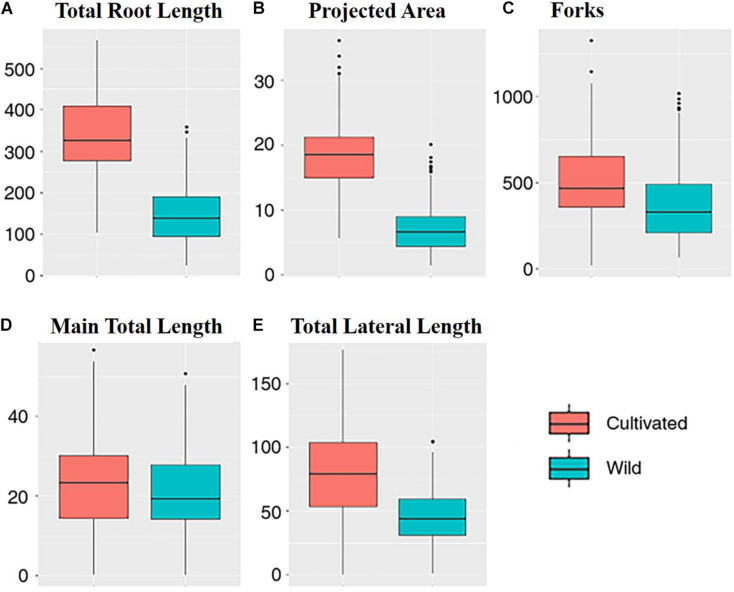
Plot of each traits: **(A)** the total root length; **(B)** the projected area; **(C)** the forks; **(D)** the main total length; and **(E)** the total lateral length (blue, wild; red, cultivated).

Between the wild and cultivated plants, there was a significant difference among five traits, including TRL, PA, FK, MTL, and TLL (Table 2). However, the *p-*value of MTL between the plants was higher (0.045) than that of others (<0.001). The values of each trait were higher in the cultivated and wild plants (Figure 2). This result could be because the above-ground biomass of the cultivated plants is significantly more than that of the wild plants. However, the MTLs of both the cultivated and wild plants were not significantly different (Table 1). This result indicates that MTL is an essential trait for survival in the wild and leads to better performance in the cultivated field.

**TABLE 2:** Kruskal–Wallis rank-sum test for wild and cultivated soybean (By Wild/Cult).

	TRL	PA	FK	MTL	TLL
Df	2	2	2	2	2
*P-*Value	<0.001	<0.001	<0.001	0.045	<0.001
chi-square	232.930	264.370	36.795	4.036	94.186

*TRL, total root length; PA, projected area; FK, forks; MTL, main total length; TLL, total lateral length.*

### Correlations

According to the correlation analysis, both soybean plant types mostly showed a significant correlation among TRL, PA, FK, MTL, TLL, and AD, except for the correlation between few traits [PA and AD (wild plants); MTL and AD (wild and cultivated plants)] (Table 3). Both plant types showed correlations between L and AD (−0.12 for cultivated plants and −0.41 for wild plants), between FK and AD (−0.19 for cultivated plants and −0.56 for wild plants), and between MTL and TLL (0.54 for cultivated plants and 0.73 for wild plants). By contrast, the correlation between TLL and AD showed a difference (Table 3). These results suggest that longer MTL means longer TLL in wild plants, whereas longer MTL does not necessarily mean longer TLL in cultivated plants. As stated above, TRL was highly correlated with PA in both cultivated (0.92) and wild (0.82) plants and relatively less correlated with MTL (0.43 for cultivated and 0.27 for wild plants) and TLL (0.82 for cultivated and 0.52 for wild plants) even though TRL comprises the sum of MTL and TLL. That tendency was particularly prominent in the wild plants rather than in the cultivated plants. The correlation between TRL and TLL was higher than that between TRT and MTL for both the cultivated and wild plants, suggesting that the length of lateral root branches is more responsible for TRL than the main root length.

**TABLE 3:** Spearman rank correlation of wild and cultivated soybean (raw data).

		TRL	PA	FK	MTL	TLL	AD
TRL	Wild	1.00	0.85***	0.83***	0.27**	0.52***	−0.41***
	Cultivated	1.00	0.92***	0.91***	0.43***	0.82***	−0.12*
PA	Wild		1.00	0.61***	0.34***	0.52***	0.08
	Cultivated		1.00	0.80***	0.42***	0.74***	0.25***
FK	Wild			1.00	0.19*	0.42***	−0.56***
	Cultivated			1.00	0.35***	0.73***	−0.19***
MTL	Wild				1.00	0.73***	0.05
	Cultivated				1.00	0.54***	0.01
TLL	Wild					1.00	−0.14*
	Cultivated					1.00	−0.14*

TRL, total root length; PA, projected area; FK, forks; MTL, main total length; TLL, total lateral length; AD, average diameter. * <0.05, ** <0.01, *** <0.001.

The correlations between TRL and MTL as well as TRL and TLL were stronger in the cultivated plants than in the wild plants, suggesting that MTL and TLL are more explanatory compared with TRL in cultivated plants than in wild plants. There was a stronger correlation between TRL and AD in the wild plants (−0.41) than in the cultivated plants (−0.12). Our results showed that the diameter of the roots is more consistent in the wild plants compared with that in the cultivated plants. Although most results showed a positive correlation among root morphological traits, only AD showed a negative correlation with TRL, FK, and TLL in both the cultivated and wild plants. The highest correlation values for the ranked comparison between TRL and PA were 0.92 (cultivated plants) and 0.85 (wild plants), respectively. The correlations between TRL and AD as well as between FK and AD were stronger in the wild plants than in the cultivated plants, whereas the correlation between FK and MTL was much stronger in the cultivated plants than in the wild plants.

### PCA

**FIGURE 3:**
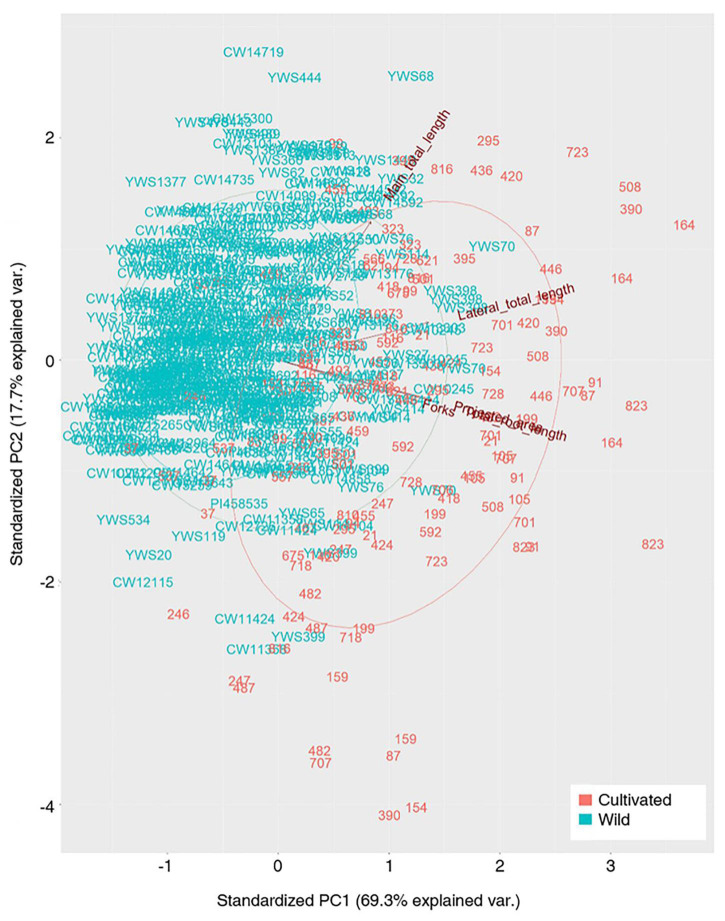
Plot of the first-two PC loading vectors (blue, wild; red, cultivated).

Principal component analysis revealed a variation between the cultivated plants and wild plants (Figure 3). The cultivated and wild plants could be separated, but there was an overlap. The variation among the cultivated plants was higher than that among the wild plants. Three groups, including MTL, TLL, and the rest of the traits, could explain all the variance. Of note, a very high variation was found among the cultivated plants, which implies a considerable breeding potential for the root traits, if necessary.

## Discussion

The root area shows the enormous diversity by several factors such as soil physical property, chemical composition of soil, soil moisture and soil nutrients (Neina, 2019). In general, however, the root area is known as over fifty percent of the whole plant area (Bardhan et al., 2021). Moreover, roots regulate or participate in various physiological mechanisms in the plant; therefore, understanding root morphological traits is crucial for plant research. In soybean research, the identification of root traits is required to expand the research field, which can help improve water and nutrient efficiency to increase productivity under uncertain water and nutrient conditions. For these reasons, many studies have been conducted to identify root morphologies in cultivated and wild plants (Costa et al., 2001; French et al., 2009; Lobet et al., 2011; Zhao et al., 2017). However, most root studies were conducted with a limited number of cultivars or germplasm. Thus, these studies could not show enough variation among and within cultivars or germplasm. This is because most of these studies collected root phenotypes manually; hence, it was challenging to show detailed root morphological traits. Therefore, we investigated root morphological traits using the image basis analysis technology to improve the limitation of previous studies. According to previous QTL studies, loci associated with root morphological traits were found to be inconsistent. Liang et al. (2014) analyzed the lateral root number, maximum root length, and root volume to identify related QTLs. They located QTLs in chromosome 2 with two loci and chromosome 4 with five loci. Prince et al. (2020) investigated QTLs that are involved in taproot length; QTLs were located in chromosome 8 with one locus and chromosome 7 with one locus. Although both studies used similar root traits, different QTLs were identified. We believed that this issue could be resolved using a large-scale dataset of root traits in many cultivars and wild germplasm, thereby enabling researchers to detect more unidentified loci.

Similar to correlation studies on root characters, many studies examined the association between under- and above-ground traits. Plant height, one of the above-ground traits, was not correlated with root length in the study by Liang et al. (2014), whereas an inconsistent result was reported by Mayaki et al. (1976). QTL studies on under- and above-ground traits were also inconsistent with respect to the location and number of loci (Liang et al., 2014; Prince et al., 2015).

Only when that information is obtained, numerous traits can be efficiently incorporated into new cultivars. Many rare alleles from wild plants have been employed to improve various agronomic traits in cultivated plants (Hajjar and Hodgkin, 2007; Liu et al., 2007). Such improvements include stress tolerance (Carter et al., 2004; Chen et al., 2006; Tuyen et al., 2010), seed compositional traits (Kanamaru et al., 2006), and seed yield (Li et al., 2008). In addition to these traits, the root traits also have been incorporated from wild plants to cultivated plants. Root length root volume are highly associated with stress tolerance (drought and aluminum stress) indices (Liu et al., 2005; Yang et al., 2005). Plants with drought tolerance ability have a profound rooting ability (Taylor et al., 1978) and more fibrous roots (Myers et al., 2007) for the effective acquisition of water, which is positively associated with improved yields under drought conditions (Hudak and Patterson, 1995). Root volume and surface area can facilitate foraging and phosphorus accumulation (Liang et al., 2014) and even improve shoot growth (Bates and Lynch, 2001). The results of the present study, however, showed that these traits were present in both wild and cultivated plants, considering the large variation found in PCA.

These root morphologies are not the only factors that are correlated with yield components. High numbers of mitochondria, Golgi bodies, and amyloplasts in root tip cells can affect shoot growth and yield (Yang et al., 2012). Furthermore, the mechanism underlying root–shoot and root–soil interactions, roles of root-sourced hormones in regulating crop growth and development, and soil moisture and nutrient management on the root architecture and physiology may be necessary for the root morphology itself. Indeed, Ramos et al. (2010) reported that root morphology changes depending on soil physicochemical characters. This interaction was supported by Forde and Lorenzo (2001) when they proposed “trophomorphogenesis” to describe changes in plant morphology that arise from variations in the availability or distribution of nutrients in the environment. They described the mechanisms of external and internal nutrient sensing, the possible nature of long-distance signals, and the role of hormones in the trophomorphogenic response. This makes sense because roots have evolved to adapt to dynamic soil conditions, including soil moisture, soil property, soil nutrition, and soil temperature (Wasaya et al., 2018). Nevertheless, the present study would be useful for “root breeding” because our study was conducted with large numbers of both the wild and cultivated plants. This finding enabled us to observe the end results of interactions in the “standard condition.” Once changes in the standard condition are understood, these changes can be evaluated in the given field condition, which does not refer to location of the wild and cultivated plants but the target location for cultivation.

According to various studies, wild plants have diverse genetic resources involved in abiotic stress resistance, including salinity (Ji et al., 2010), drought (Kao et al., 2006), flooding (Li et al., 2017), and high temperature (Nawaz et al., 2018). Thus, they are considered as an alternative to breeding new cultivars because they contribute a significant proportion of genetic resources to mitigate unfavorable environmental conditions for cultivation (Dwivedi et al., 2017; Zhang et al., 2017; Mammadov et al., 2018). Moreover, the root may evolve as per the given environment, which could be vast sources for breeding. One of the most important root features to measure for breeding is its morphology. The data obtained in this study by the phenome technology provided a better estimation of the correlations among traits because of the large numbers of cultivated and wild plants. More importantly, our results revealed huge variations in root morphologies even among cultivated plants, which is sufficient to break the general myth “screen wild germplasm for new traits.” Indeed, the study results showed a huge potential of root breeding in soybean. In the future, it is suggested that the root shape should be examined using the method by Falk et al. (2020) to gain an insight in the variation of the root shapes as well as the traits examined in the present study.

## Conclusion

We analyzed numerous images of the roots of cultivated and wild soybean using WinRHIZO software. We revealed that there is a huge variation in root morphology. Of note, we found that the variation is larger in cultivated than in wild plants. Furthermore, we found the following relationships among root traits. First, TRL significantly decides PA in the wild and cultivated plants. Second, TRL is more affected by FK in both the wild and cultivated plants. Third, TRL in wild plants is shorter than that in cultivated plants, and FK is less developed as AD increases, unlike in cultivated plants. Fourth, TLL in cultivated plants is highly affected by TRL, PA, and FK; however, in wild plants, it is positively associated with MTL. There was no correlation between MTL and AD, suggesting that these two traits independently segregate each other. However, a high correlation found in this study could imply two characteristics: linked and co-selected. One trait can be selected by the other trait when they are highly correlated and linked; however, if they are not correlated and linked, the traits are co-selected, which can be discovered by a molecular marker study. Taken together, our results showed that there is enough room for root breeding, particularly in cultivars.

## Data Availability Statement

The original contributions presented in the study are included in the article/Supplementary Material, further inquiries can be directed to the corresponding author/s.

## Author Contributions

K-SK, S-HK, and JK wrote the manuscript and analyzed the root phenotype data. PT participated in PCA analysis. J-DL assisted with the data collection and donated wild soybean germplasm. YC and YK inspected the experimental design and revised the manuscript. All authors contributed to the article and approved the submitted version.

## Conflict of Interest

K-SK was employed by the company FarmHannong Ltd. The remaining authors declare that the research was conducted in the absence of any commercial or financial relationships that could be construed as a potential conflict of interest.

## Publisher’s Note

All claims expressed in this article are solely those of the authors and do not necessarily represent those of their affiliated organizations, or those of the publisher, the editors and the reviewers. Any product that may be evaluated in this article, or claim that may be made by its manufacturer, is not guaranteed or endorsed by the publisher.

## Supplementary Material

Supplementary Table 1 List of soybean genotypes.

Supplementary Table 2 Description of root morphological traits used in the study by WinRHIZO software.
