# Supplementary material for: A Large Root Phenome Dataset Wide-Opened the Potential for Underground Breeding in Soybean
Source: Front Plant Sci. 2021 Aug 5;12:704239. doi: 10.3389/fpls.2021.704239 (PMC8374737; doi:10.3389/fpls.2021.704239)
Supplement: Supplementary Table 1 — List of soybean genotypes. [file Table_1.docx]

**Supplement Table 1.** List of soybean genotypes

| **Wild soybean** | | | | | | **Cultivated soybean** | | |
| --- | --- | --- | --- | --- | --- | --- | --- | --- |
| **Genotype** | **IT number** | **Genotype** | **IT number** | **Genotype** | **IT number** | **Variety** | | **IT number** |
| YWS62 | - | YWS101 | IT 247456 | CW14573 | - | Kirby | 165087 | |
| PI468400A | - | CW10262 | - | CW11424 | - | Simpson | 165132 | |
| YWS1411 | - | CW14719 | - | CW14708 | - | Kwangan | 199092 | |
| CW14090 | - | YWS564 | - | CW14657 | - | PI 467330 | 171092 | |
| CW11355 | - | YWS876 | - | YWS122 | - | Yantarnaya | 199092 | |
| YWS55 | IT 247444 | CW11944 | - | YWS444 | - | Pixie | 24681 | |
| CW12726 | - | PI424096 | - | YWS443 | - | Damahigari | 165221 | |
| CW14580 | IT 267433 | YWS587 | IT 247542 | YWS4 | IT 250557 | Ogden | 22773 | |
| CW13452 | - | CW14748 | - | CW13168 | - | Stafford | 165136 | |
| YWS85 | IT 247453 | CW15263 | - | YWS651 | - | Forrest | 23532 | |
| YWS52 | - | YWS80 | - | CW14604 | IT 270014 | PI 464879 | 165217 | |
| YWS27 | - | YWS39 | - | YWS76 | - | PI464922 | 165255 | |
| CW14642 | IT 270016 | CW14691 | - | CW14732 | - | Macoupin | 22721 | |
| YWS618 | - | YWS1334 | - | YWS18 | IT 242674 | Jackson | 22341 | |
| CW14643 | IT 267444 | YWS681 | - | YWS54 | IT 250554 | 165295 | 22721 | |
| CW11358 | - | YWS522 | IT 250417 | YWS398 | - | Hawkeye | 21641 | |
| YWS119 | - | CW13143 | - | YWS262 | IT 247464 | Hujimijiro | 180396 | |
| YWS567 | - | CW15260 | - | CW11929 | - | PI 458523 | 165192 | |
| YWS1449 | - | PI458535 | IT 283576 | CW14628 | - | Oakland | 24367 | |
| YWS68 | - | YWS490 | - | YWS32 | - | Hei nong 35 | 196407 | |
| CW13701 | - | CW15259 | - | CW14648 | - | Ross | 23093 | |
| CW14449 | - | CW14651 | IT 267445 | YWS70 | IT 250553 | Hoemok Gwandong 101 | 181031 | |
| CW11260 | - | CW14750 | - | CW14636 | IT 267443 | Maple Glen | 165097 | |
| CW14667 | - | YWS8 | - | CW13821 | - | Jungdu 8 | 180429 | |
| CW14635 | - | CW14711 | - | CW10023 | - | Nasushirome | 22754 | |
| CW11907 | - | CW14099 | - | YWS1461 | - | Beltskaya 25 | 199095 | |
| CW10236 | - | CW13176 | - | YWS179 | - | Dankyeonkong | 154764 | |
| YWS1382 | - | PI479750 | - | CW15265 | - | PI 464897 | 165231 | |
| YWS360 | - | YWS692 | IT 247560 | YWS585 | - | Mokapu summer | 24364 | |
| YWS627 | - | YWS432 | - | YWS488 | - | Wayne | 21812 | |
| CW14721 | - | YWS512 | IT 242678 | CW11782 | - | PI 466749 B | 165263 | |
| YWS270 | - | CW13185 | - | YWS1406 | - | Zhong huang 6 | 211824 | |
| YWS60 | - | CW13313 | IT 267426 | YWS31 | - | FC32176 | 160790 | |
| YWS114 | - | CW14661 | - | CW14644 | - | Gongjiao 5610-1 | 165841 | |
| YWS433 | - | CW14104 | - | YWS414 | - | Bokwang | 154767 | |
| CW14858 | - | YWS11 | - | CW14722 | - | Eif | 170861 | |
| CW13455 | - | CW14735 | - | CW14726 | - | PI 479742 | 165437 | |
| PI407296 | IT 236853 | CW14769 | - | YWS103 | - | HARDIN | 142750 | |
| CW10852 | - | CW14669 | - | YWS1400 | IT 242875 | PI479712 | 165405 | |
| CW10245 | - | YWS1405 | - | YWS20 | IT 247441 | Vance | 165160 | |
| CW14682 | - | YWS399 | - | CW14694 | - | Lawrence | 170900 | |
| YWS50 | - | CW11550 | - | YWS1379 | - | Belor | 208349 | |
| CW12964 | IT 267416 | YWS1377 | - | CW12101 | - | Gnome | 165060 | |
| YWS622 | - | YWS534 | - | YWS489 | - | Young | 165162 | |
| CW14631 | IT 267441 | CW14675 | - | YWS1 | - | PI467311C | 165271 | |
| CW13303 | - | CW13256 | - | YWS470 | - | Shinsey | 23121 | |
| CW12115 | - | PI483461 | IT 236885 | CW14670 | - | Jupiter-R | 163447 | |
| CW14428 | - | YWS65 | - | CW15300 | - | Damahigari | 100868 | |
| YWS34 | - | CW14592 | IT 270013 | CW12420 | - | Guan dou 1 | 212822 | |
| CW10915 | - | YWS597 | - | CW14654 | - | PI467321 | 165284 | |

**Supplement Table 2.** Description of root morphological traits used in the study by WinRHIZO software

| Root morphological traits | Description |
| --- | --- |
| Total root length (TRL) | Length of root skeleton, counting pixels while considering the direction of root displacement |
| Projected area (PA) | The area occupied by roots in the image by counting the number of pixels belonging to the root in the detected root images then multiplying by the pixel area |
| Average diameter (AD) | Mean diameter of roots |
| Forks (FK) | Number of new branchings |
| Main total length (MTL) | The total length of links of order 0 (main) |
| Total lateral length (TLL) | The total length of links of order 1 (lateral) |
